# The role of lipids in the effect of *APOE2* on cognitive function: a causal mediation analysis

**DOI:** 10.1007/s10654-025-01310-0

**Published:** 2025-10-03

**Authors:** Qingyan Xiang, Judith J. Lok, Nicole Roth, Stacy L. Andersen, Thomas T. Perls, Zeyuan Song, Anatoli I. Yashin, Jonas Mengel-From, Gary J. Patti, Paola Sebastiani

**Affiliations:** 1 Department of Biostatistics, Vanderbilt University Medical Center, Nashville, TN 37235, USA; 2 Department of Mathematics and Statistics, Boston University, Boston, MA, USA; 3 Section of Geriatrics, Department of Medicine, Boston University Chobanian & Avedisian School of Medicine, Boston, MA, USA; 4 Institute for Clinical Research and Health Policy Studies, Tufts Medical Center, Boston, MA, USA; 5 Biodemography of Aging Research Unit, Social Science Research Institute, Duke University, Durham, NC, USA; 6 Department of Public Health, University of Southern Denmark, Odense, Denmark; 7 Department of Genetics, Washington University in St. Louis, St. Louis, MO, USA; 8 Department of Chemistry, Washington University in St. Louis, St. Louis, MO, USA; 9 Department of Medicine, Washington University in St. Louis, St. Louis, MO, USA; 10 School of Medicine, Tufts University, Boston, MA, USA; 11 Data Intensive Study Center, Tufts University, Boston, MA, USA

**Keywords:** Apolipoprotein E, Cognition, Longevity, Mediation analysis, Observational data

## Abstract

Extensive research has examined the direct effect of *APOE* alleles on cognitive decline. However, there is limited investigation into the effect of *APOE* that is explained or mediated through molecular pathways, such as lipids. In this study, we performed a causal mediation analysis to estimate both the direct effect of *APOE2* and its indirect effect through 24 lipid species on cognitive function, measured from the digital Clock Drawing Test (CDT) in 1228 Long Life Family Study (LLFS) participants. Results showed that *APOE2* carriers completed the CDT significantly faster compared to common *APOE3* carriers. Primary analysis identified two lipids (CE 18:3 and TG 56:5) protectively mediated the effect of *APOE2* on cognitive function, resulting in shorter CDT think-time, ink-time, and total-time; conversely, TG 56:4 deleteriously mediated the effect of *APOE2*, resulting in increased ink-time. Secondary analysis yielded consistent results and identified four additional significant lipid pathways (DG 38:5, TG 51:3, TG 56:1, TG 56:2) that mediated the effect of *APOE2*. The combined indirect effect in the primary analysis contributed 15%–30% mediated proportion on CDT times, though such mediated proportion did not reach statistical significance. Overall, our analysis identified seven lipid species that significantly mediate the effect of *APOE2* on cognitive performance. These lipids represent distinct lipid pathways, including both protective and deleterious mediation effects. Our findings offer insights for new therapeutics targeting those lipids to enhance the protective effects of *APOE2* on cognition.

## Introduction

The apolipoprotein E (*APOE*) gene, a crucial gene in lipid metabolism, has been extensively studied for its association with cognitive function and late-onset Alzheimer’s Disease (AD) [1–4]. The *APOE* gene has three well-characterized alleles—e2, e3, and e4—that are defined by combinations of the Single Nucleotide Polymorphisms (SNPs) rs7412 and rs429358. Among these alleles, the e3 allele is the most common in Non-Hispanic and White individuals and is considered neutral. The e4 allele is considered as a major genetic determinant for AD risk and cognitive decline [5–7], and the e2 allele is associated with increased human longevity [8] and decreased risk for AD and cognitive decline [9–13].

Extensive research has focused on the direct effect of *APOE* alleles on the risk for AD and cognitive decline. However, there have been limited investigations on the effect of *APOE* that is explained or mediated through molecular pathways [14–16], such as lipids. *APOE* plays an important role in lipid metabolism, as the previous research showed that different *APOE* alleles are associated with replicated lipid profiles [14, 17, 18]. Compared to genes, lipids are modifiable risk factors for cognitive decline [19]. Therefore, characterizing the role of lipids as mediators can improve our understandings of the mechanism of *APOE* on cognition, which provides insights for developing new therapeutics that target these lipid pathways.

In this study, our objective is to investigate the effect of *APOE2* on cognitive function and to examine whether this effect is mediated through lipid metabolites. We analyzed the data from Long Life Family Study (LLFS) [20], an observational study of families of participants with exceptional longevity, whose cognitive function is assessed by the Clock Drawing Test (CDT). The CDT is a widely used screening tool for global cognitive dysfunction, where faster CDT completion time have been associated with better processing speed and logical memory [21]. Three metrics were derived from the digital version of CDT: think-time, inktime, and their sum as total-time. Focusing on a list of 24 lipids previously identified as significantly associated with *APOE2* [22], we performed a causal mediation analysis to estimate (1) the direct effect of *APOE2*, (2) the indirect effects of *APOE2* through these lipids, and (3) the total effect of *APOE2* on CDT think-, ink-, and total-time among 1228 LLFS participants.

## Methods

### Participants

#### Long Life Family Study (LLFS)

The LLFS is a multicenter, multigeneration study that enrolled 4,953 family members from 539 families who exhibit healthy aging and longevity. Participants were first enrolled between 2006 and 2009 at three American field centers (in Boston, Pittsburgh, and New York) and a Danish field center. The second in-person visit was completed during 2014–2017 for participants using the same protocols. Further details on the LLFS study can be found in reference [20]. All participants provided informed consent through their local Institutional Review Board, and the genetic and phenotypic data generated through 2017 are available through dbGaP (dbGaP Study Accession: phs000397. v1.p1). New data generated after 2017 are distributed through the ELITE portal: https://eliteportal.synapse.org/Explore/Projects/DetailsPage?shortName=LLFS.

#### APOE genotype data

*APOE* alleles were determined from genotypes of two SNPs, rs7412 and rs429358, that were generated using Whole Genome Sequencing [20]. The e2 allele was defined by the combination rs7412=T and rs429358=T; the e3 allele was defined by the combination rs7412=C and rs429358=T; and the e4 allele by rs7412=C and rs429358=C. This study focused on the comparison between genotype group *APOE3* versus genotype group *APOE2* in LLFS participants, where the genotype groups were defined as: *APOE3*=e3e3 (reference group) and *APOE2*=e2e2 or e2e3. Carriers of one or more copies of the e4 alleles were excluded.

#### Lipid

For lipid measurements, this study used blood collected during the first in-person visit between 2006 and 2009. Lipids were analyzed from plasma by liquid chromatography/mass spectrometry (LC/MS) as described previously [17, 22]. Lipids were first isolated by using solid-phase extraction kits. Then, they were separated by reversed-phase chromatography prior to being measured on an Agilent 6545 quadrupole time-of-flight mass spectrometer at Washington University in St Louis. Samples were analyzed in batches of approximately 90. Pooled samples, reference materials, and internal standards were used for quality control and batch correction, thereby ensuring high data quality. We processed the data with a combination of XCMS, DecoID, and Skyline to facilitate removal of background, annotation of adducts, and compound identification [24, 25]. We normalized data by using a random forest-based approach for batch correction which we previously found to be the optimal approach for this data [26]. We identified lipids based on retention time and fragmentation pattern matches both in-house and public databases formed from analysis of authentic standards. For retention time matches, we allowed a 30s tolerance between reference and observed retention times. For MS/MS matches, a normalized dot-product score of >80 was required. We reviewed all identifications manually after applying the cutoffs listed previously to ensure accurate matches. According to the Metabolomics Standard Initiative [27], these identifications correspond to high-confidence Level 1 and Level 2 identifications. A detailed description of these methods was described in a prior report [28].

In this analysis, we used 24 lipids that were associated with *APOE2* at 5% false discovery rate, as reported in Sebastiani et al. (2024) [22]. Supplementary Table 1 includes the estimated association between *APOE2* and each lipid, as well as adjusted p-values. All lipids were log-transformed and standardized for the mediation analysis.

#### Clock Drawing Test (CDT)

The CDT is a common screening test for global cognitive dysfunction and for a range of neurological and psychiatric illnesses [29]. It was added to the neuropsychological assessment protocol at the second in-person assessment and administered using a digital pen that recorded spatial-temporal features of the test performance. Participants were given a piece of specially formatted paper that can be read by the digital pen folded down to 8 by 5.5 inches. In the Command Condition the examiner read the instructions “I’d like you to draw a clock, put in all of the numbers, and set the hands to ten after eleven.” Immediately following, the Copy Condition was administered. On the other side of the paper, a picture of a clock with the hands set to 11:10 was displayed and the examiner instructed “Please copy this clock.” Each condition was discontinued if the participant was unable to complete their drawing within 5 min. Only data from the more cognitively demanding Command Condition were used in this analysis.

Digital features of the CDT were extracted by digital software developed by Massachusetts Institute of Technology and Lahey Hospital and Medical Center [30]. *Thinktime*, the time spent holding the pen without drawing, was conceptualized to capture cognitive processing while the participant is planning the next component to draw [31]. *Ink-time*, the time spent drawing on the paper, was developed to capture motor aspects of test performance. In a separate study [32], thinking time and ink time were extracted from a coding test and found to correlate with cognitive processing and motor function, respectively, in line with their conceptualization. We also computed an additional metric of total-time using the sum of think-time and ink-time.

### Statistical analysis

**Fig. 1:**
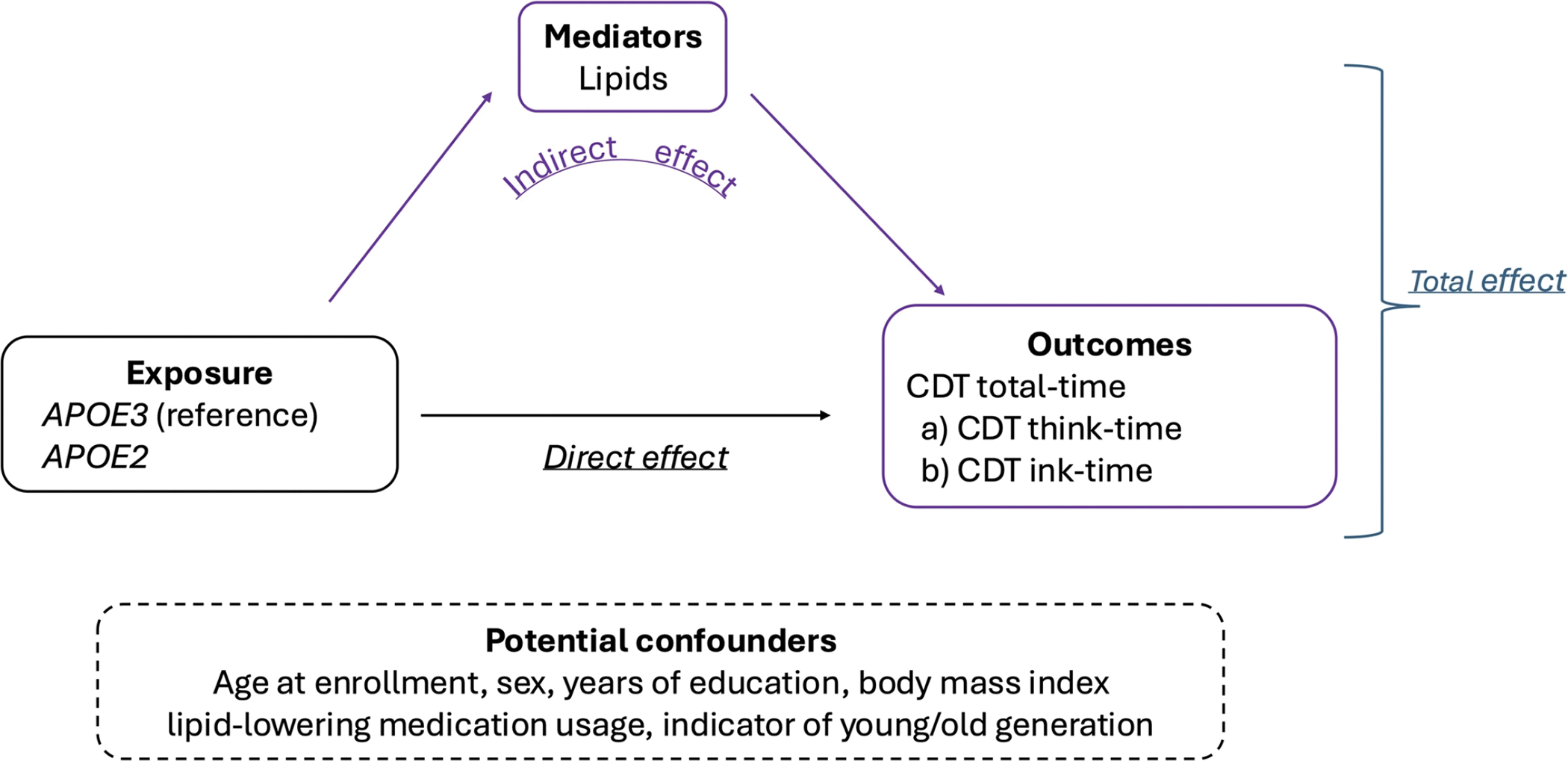
Components of the mediation analysis in this study: exposure, mediators, potential confounders, and outcomes. *CDT* Clock Drawing Test

To study how the effect of *APOE* on cognition is mediated by lipids, we performed a causal mediation analysis to estimate the direct and indirect effects of *APOE2* on CDT performance. The causal mediation analysis (Fig. 1) included these primary components:

- *Exposure*: *APOE2* genotype group *versus APOE3* genotype group (reference group).
- *Mediators*: Lipids level data that were log-transformed and then standardized.
- *Confounders measured at baseline*: age at enrollment, sex, education, body mass index (BMI), lipid-lowering medication usage, and indicator of young/old generation based on whether the birth year > 1935 (see Supplementary Fig. 1 for the distribution of birth year in LLFS participants).
- *Outcomes*: CDT total-time derived by summing (a) CDT think-time and (b) CDT ink-time.

In this analysis, the lipids (mediators) are measured at the first visit and the CDT results (outcomes) are measured at the second visit, satisfying the temporal ordering assumption of the mediation analysis, where the causal sequences follow exposure →mediator →outcome.

We used a regression-based approach for the causal mediation analysis with multiple mediators, as described in references [33, 35]. First, we fit the *mediator regression model* for each of the lipids, where the independent variable was the *APOE* genotype group, adjusted for all the confounders. Next, we fit the *outcome regression model* for each CDT outcome separately, where the independent variables were the *APOE* genotype group and all lipids simultaneously, adjusted for all the confounders. Then, we combined the estimates from both *mediator* and *outcome* regression models [33] to estimate the direct effect and indirect effect of *APOE2* on the CDT times. We include the modeling details for this mediation analysis in the supplementary file. In addition, to account for within-family correlations, we used generalized estimating equations to estimate standard errors in all regression models with an exchangeable covariance matrix based on family IDs.

We also performed a secondary analysis including a subset of lipids instead of all lipids. We applied a backward stepwise variable selection in the outcome regression model based on the Akaike Information Criterion (AIC) to select lipids. We then repeated the entire causal mediation analysis only including the lipids retained in the regression models after variable selection. Finally, we performed a sensitivity analysis by excluding the variable of lipid-lowering medication usage in the models, and we repeated the mediation analysis.

In reporting the mediation analysis results, we present:

1. *Direct effect* of *APOE2* on the CDT times.
2. *Individual indirect effect* mediated through each lipid, where *APOE*2 affects each lipid, which in turn affects the CDT time.
3. *Combined indirect effect*, the sum of all individual indirect effects via lipid pathways.
4. *Total effect*, the sum of the direct effect and the combined indirect effect.
5. *Mediated proportion*, the proportion of total effect that is mediated through all lipid pathways, that we calculated as the combined indirect effect divided by the total effect.

We generated 95% confidence intervals (CI) for these effect estimates using the bootstrap with Efron’s percentile method [35] using 1500 replicates. Whether a 95% CI includes zero will be used to assess the statistical significance. We used R 4.1.3 for all analyses and all scripts are available from QM&DS Tufts Medical Center github (https://github.com/QM-DS-Tufts-Medical-Center).

## Results

### Participant and lipid characteristics

Our analysis included 1228 participants with *APOE* genotype data and plasma lipids measured at the first visit and CDT data from the second visit. Table 1 summarizes the characteristics of the LLFS participants included in this analysis. Among those participants, *APOE3* carriers and *APOE2* carriers had similar ages at enrollment, years of education, and BMI. However, *APOE3* carriers included a higher proportion of females (58% versus 54%) and those who takes lipid lowering medications (32% versus 18%), compared to *APOE2* carriers.

Table 2 lists the 24 lipid species that were included in the analysis. These lipids include sterol lipids (CEs), sphingolipids (DGs), glycerolipids (TGs), dHexCer_NS 34:1, dHexCer_NS 40:1, dHexCer_NS 41:1, and dHexCer_NS 42:1.

### Primary analysis of digital CDT.

#### Mediator regression and outcome regression

Supplementary Table 2 shows the results of the mediator regression that describes the associations between *APOE2* and lipids (log scale and standardized). Consistent with previous work [17], the estimated associations between *APOE* and the 24 lipids were almost all statistically significant. Supplementary Table 3 shows the results of the outcome regression, which shows that *APOE2* had a statistically significant protective association that leads to reduced total-, think-, and ink-time, after adjusting for all lipids and confounders. Among all the lipids, CE 18:3 was positively associated with think-time (*β* = 1.07, *p* = 0.03); TG 56:4 was positive associated with ink-time (*β* = 1.06, *p* = 0.03); and TG 56:5 was negatively associated with total-time (*β* = −4.37, *p* = 0.05) and ink-time (*β* = −1.60, *p* < 0.01). No other lipid species was significantly associated with any of the CDT times.

#### Individual indirect effect of APOE2 on CDT times through lipids

**Fig. 2:**
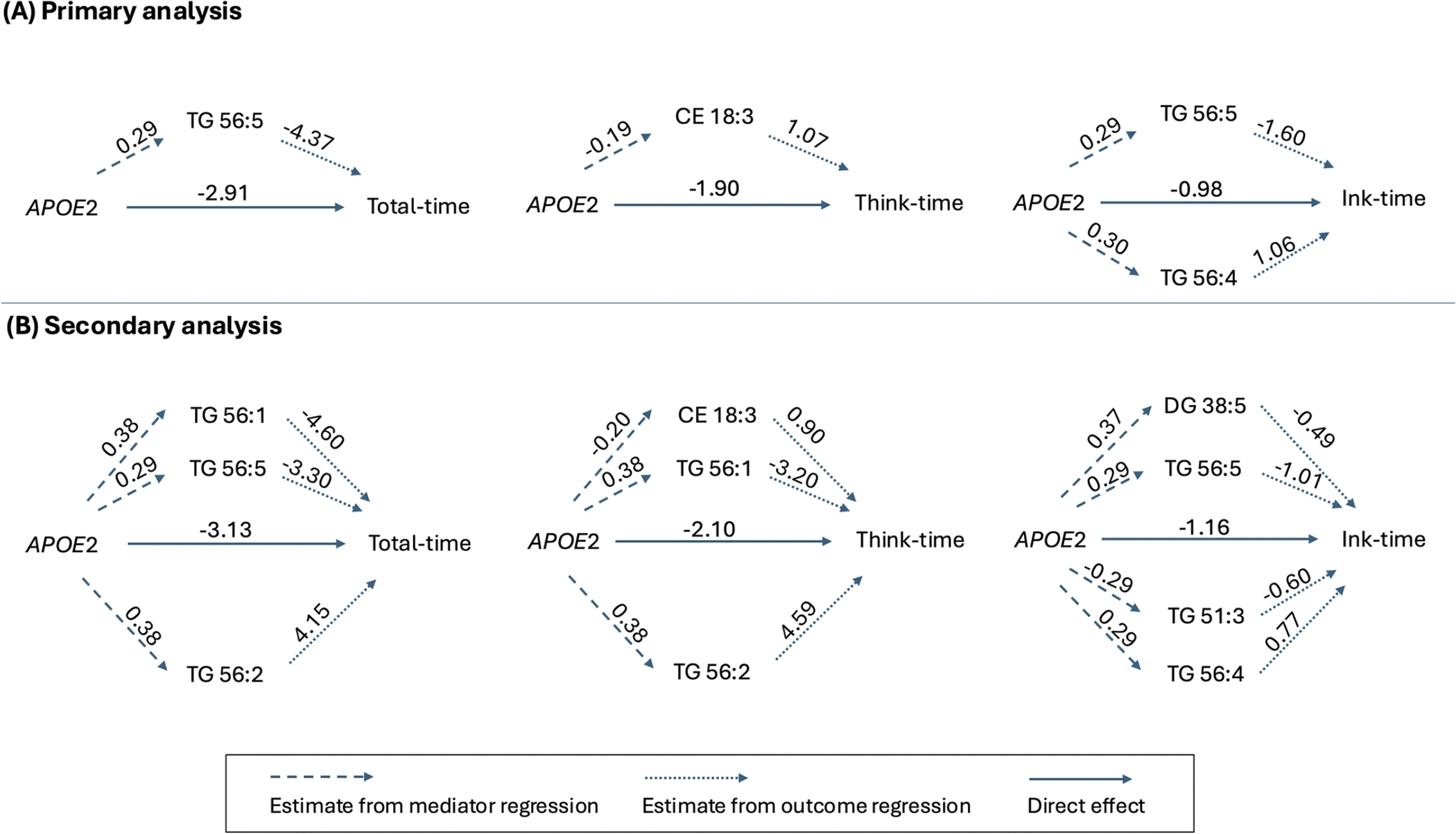
Mediation analysis results: significant direct and indirect effects in primary analysis (**A**) and secondary analysis (**B**). The numbers on the dashed lines represent the estimated associations between *APOE2* and the lipids from mediator regression, and on the dotted lines represent the estimated associations between the lipids and the CDT times from outcome regression. The solid lines represent the direct effect of *APOE2* on CDT times. Protective lipids reducing CDT times are above the solid arrow in the middle, e.g., CE 18:3; Deleterious lipids increasing CDT times are below the solid arrow in the middle, e.g., TG 56:4

**Fig. 3:**
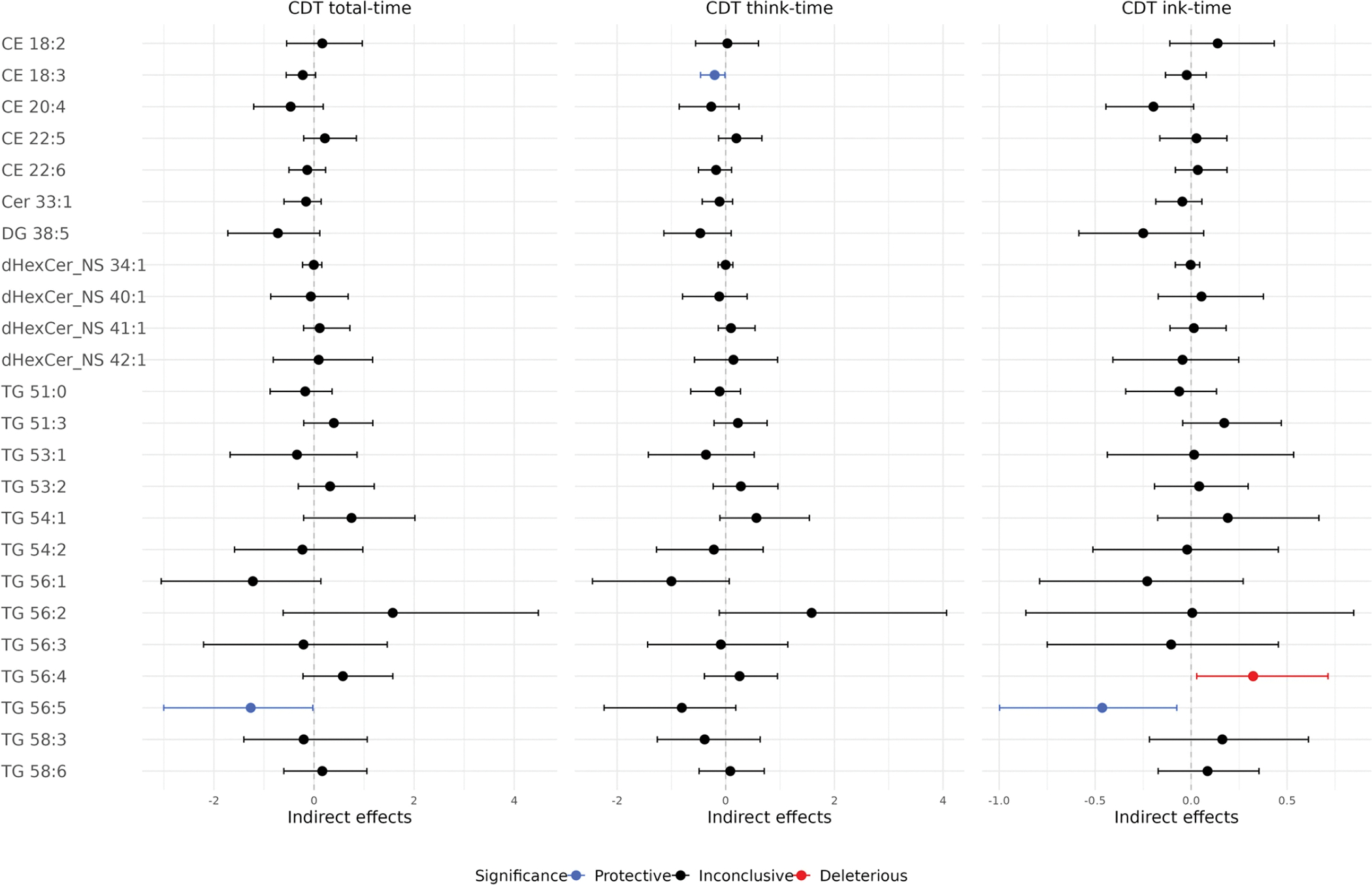
Primary analysis of indirect effects of *APOE2* on CDT times through each lipid pathway. Blue color indicates statistically significant protective lipids, which mediated the effect of *APOE2* to reduce CDT times. Red color indicates statistically significant deleterious lipids, which mediated the effect of *APOE2* to increase CDT times

Figure 2A shows the significant direct effect of *APOE2* and the significant indirect effects through lipid-mediated pathways. Figure 3 and Table 3 show all individual indirect effects of *APOE2* on the CDT times through each lipid pathway. Note that if the estimate of an indirect effect is negative (< 0), it indicates a *protective* indirect effect of the lipid, as this pathway mediate the effect of *APOE2* to reduce the CDT time. Conversely, a positive estimate indicates a *deleterious* effect. For example, regarding the total-time in Fig. 2A, compared to *APOE3*, *APOE2* was associated with increased TG 56:5, but increased TG 56:5 led to a reduction in total-time. Therefore, the effect of *APOE2* on cognitive function was partially mediated through this protective pathway of *APOE2* → TG 56:5 → total-time.

From Fig. 3 and Table 3, three lipid species appeared to significantly mediate the *APOE2* effects: CE 18:3, TG 56:5, and TG 56:4. For the protective pathways, the indirect effect of *APOE2* through CE 18:3 reduced think-time by 0.2 s (estimate: −0.20; CI: −0.46, −0.01). The indirect effect through TG 56:5 significantly reduced total-time by 1.26 s (estimate: −1.26; CI: −3.00, −0.02) and significantly reduced ink-time by 0.46 s (estimate: −0.46; CI: −1.00, −0.07). For the deleterious pathway, the indirect effect of through TG 56:4 significantly increased ink-time by 0.32 s (CI: 0.03, 0.71).

#### Total and combined effects of APOE2 on CDT times

Table 4 summarizes the overall results of the mediation analysis. Compared to *APOE*3, *APOE*2 carriers completed the CDT test 3.98s faster in total-time (estimate: −3.98; CI −6.33, −1.36). This reduced time can be decomposed into a significant direct effect of 2.91s (estimate: −2.91; CI: −5.26, −0.59) and an insignificant combined indirect effect through all lipids of 1.07s (estimate: −1.07; CI: −2.63, 0.58); Though not significant, the combined indirect effect contributed to 27% (−13%, 82%) mediated proportion.

Consider the two components of total-time. First, the think-time of *APOE2* carriers was 2.78s faster than *APOE3* carriers (estimate: −2.78; CI: −4.80, −0.69). This reduced time can be decomposed into a direct effect of 1.90s (estimate: −1.90; CI: −3.60, −0.22) and a combined indirect effect mediated through all lipids of 0.89s (estimate: −0.89; CI: −2.28, 0.42; mediated proportion: 32%). Second, the ink-time of *APOE2* carriers was 1.15s faster than *APOE3* carriers (estimate: −1.15; CI: −2.02, −0.23). This reduced time can be decomposed into a direct effect of 0.98s (estimate: −0.98; CI: −1.80, −0.18) and a combined indirect effect of 0.17s (estimate: −0.17; CI: −0.68, 0.37; mediated proportion: 15%).

### Secondary and sensitivity analysis

#### Secondary analysis

For each CDT outcome, we performed a variable selection of lipids using the outcome regression model, and we repeated the entire causal mediation analysis using the lipids that remained in the final selected model. Importantly, after variable selection, each CDT outcome model can include different numbers of final lipids. Results of the mediator regression and the outcome regression in the secondary analyses for these CDT times are shown in Supplementary Tables 4–5.

**Fig. 4:**
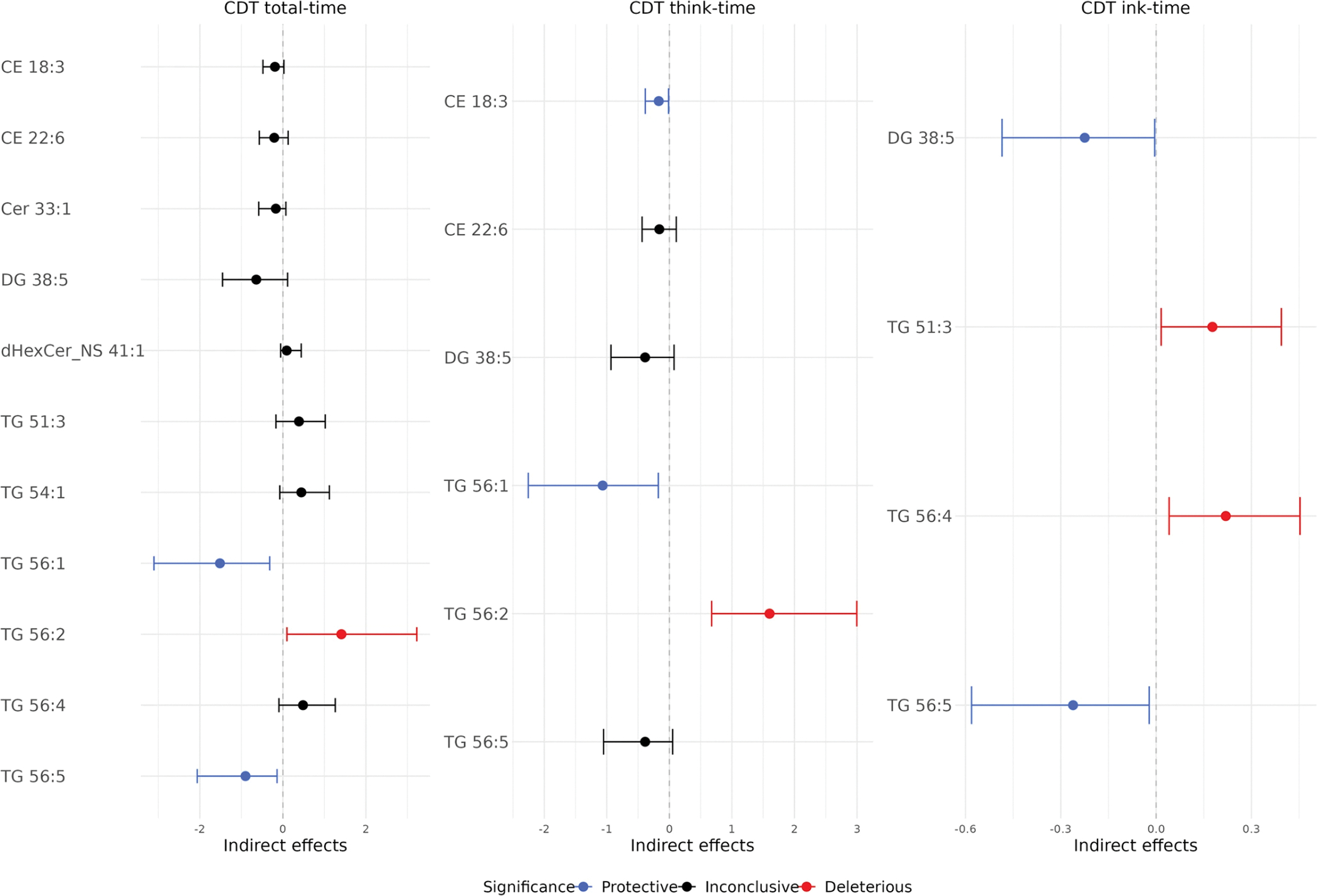
Secondary analysis of indirect effects of *APOE2* on CDT times through each lipid pathway. Stepwise variable selection was applied to select lipids, so each CDT time had different lipids remained in the mediation analysis. Blue color indicates statistically significant protective lipids, which mediated the effect of *APOE2* to reduce CDT times. Red color indicates statistically significant deleterious lipids, which mediated the effect of *APOE2* to increase CDT times

Figure 2B shows the significant indirect effects through lipid-mediated pathways in the secondary analysis. Figure 4 and Table 5 show all indirect effects of *APOE2* on the CDT times through each lipid pathway. Consistent to the primary analysis, TG 56:5 and CE 18:3 remained as protective mediators, and TG 56:4 remained as a deleterious mediator. In addition, four new lipids were also found to significantly meditate the *APOE* effects: DG 38:5 and TG 56:1 have a protective effect; TG 51:3 and TG 56:2 have a deleterious effect.

Table 5 also summarizes the results of the mediation analysis of this secondary analysis for CDT times. The total effect of *APOE2* on every CDT outcome in the secondary analysis is very similar to the primary analysis. The combined indirect effects show slight changes in magnitude, though they remain statistically non-significant. The mediated proportion of the combined indirect effect decreased across all CDT outcomes. For instance, for CDT total time, the mediated proportion decreased from 27% in the primary analysis to 20% in the secondary analysis. This is possibly due to fewer lipids remaining in the model after variable selection.

### Sensitivity analysis

For each outcome, we excluded the variable of lipid-lowering medication usage in both the mediator and outcome regression model, and we repeated the entire causal mediation analysis including all lipids. Supplementary Fig. 2 and Supplementary Table 6 show all indirect effects of *APOE2* on the CDT times through each lipid pathway in the sensitivity analysis. The results were comparable to the primary analysis. CE 18:3, TG 56:4, and TG 56:5 stayed as significant lipid pathways that mediated the effect of *APOE2*, and the only difference is that their CI become slightly narrower.

## Discussion

### Overview

Our analysis investigates the potential mediating role of lipids on the effect of *APOE2* on CDT total-time, think-time, and ink-time. In the primary analysis, we identified a significant protective direct effect of *APOE2* on CDT total-time, think-time, and ink-time, significant protective indirect effects through two lipids (CE 18:3 and TG 56:5), and a significant deleterious indirect effect through one lipid (TG 56:4). Compared to *APOE3*, the combined indirect effect of *APOE2* through all lipid pathways mediated 27% of the total effect on CDT total-time for a 1.07s faster completion time, but such mediated proportion does not reach statistical significance. Additionally, the secondary analysis revealed significant protective effects through DG 38:5 and TG 56:1, as well as significant deleterious effect through TG 51:3 and TG 56:2. Compared to the primary analysis, the total effect on all CDT times in the secondary analysis were similar, but the combined indirect effect on total-time and think-time contributed to a slightly lower mediated proportion of the total effect.

### Discussion

Although the role of *APOE* in aging and cognition has been extensively studied [1], the mechanisms by which the *APOE2* allele protects against cognitive decline and promotes longevity remain elusive [36]. The strong correlations between *APOE2* alleles and many lipid species suggest that lipids in the blood may mediate the genetic effect of *APOE* on cognitive function [14, 15, 17], where individuals with certain lipid profile can present “reduced/increased” risk of cognitive decline. Our findings in the primary and secondary analyses identified seven lipids (CE 18:3, DG 38:5, TG 51:3, TG 56:1, TG 56:2, TG 56:4, TG 56:5) that significantly mediated the effect of *APOE2*, highlighting their potential as therapeutic targets for preserving cognitive function during aging. Specifically, our analyses suggest two potential strategies for therapeutic intervention: increasing the levels of lipids that were identified as protective pathways (CE 18:3, DG 38:5, TG 56:1, TG 56:5) or decreasing the levels of lipids that were identified as deleterious pathways (TG 51:3, TG 56:2, TG 56:4).

Our analysis showed that lipids within the same super class can significantly mediate the effects of *APOE2* on cognitive function in different directions. For example, we identified glycerolipids with opposite effects. TG 56:5 showed protective mediation in both primary and secondary analysis, while TG 51:3, TG 56:2, and TG 56:4 showed deleterious mediation. These findings can help clarify TG’s complex mechanisms on cognitive function and cognitive test performance, as similar conflicting associations were also reported in previous studies [37–39]. Furthermore, the selective effects of these glycerolipids on total-time and ink-time of the CDT but not on think-time, suggest that they mediate graphomotor function components of CDT test performance more specifically.

The sterol lipids included in this analysis consistently showed their protective mediation of the effect of *APOE2*. CE 18:3 was protective for think-time in both the primary analysis and the secondary analysis. This is aligned with recent research reporting that cholesterols are either not associated with or may even protect against late-life cognitive decline [40–42]. Furthermore, the relationship of the sterol lipids with think time, rather than ink time, also suggests that they mediate cognitive processing components of CDT test performance more specifically.

Previous studies on mediation analysis of *APOE* have focused on mediators of cerebral blood flow [43], brain tissue volume [44], and neuropathological pathways [45], suggesting these factors partially mediated the negative effect of *APOE4*. When considering lipids as mediators, one study found that total cholesterol negatively mediated the effect of *APOE2* on cognition [15]; another study found no lipids but BMI significantly mediated the risk of AD [16]. In addition, one study on the risk of AD found 11 lipid species that mediated the effect of *APOE2*, accounting for up to 30% of the total effect of *APOE2* on AD resilience [14]. This is aligned with our finding of up to 32% mediated proportions from all lipid pathways. Compared to previous research, the novelty of our research lies in revealing both protective and deleterious pathways mediated through lipids, as well as differential effects of glycerolipids and sterol lipids on graphomotor function and cognitive processing, respectively.

### Limitations

In our primary analysis, the CIs for some lipids were marginally close to zero. For example, the upper 95% CI for DG 38:5 was just above zero for all CDT times in the primary analysis. The number of LLFS participants who completed the CDT are not large, which may have led to limited statistical power in those findings. Future research may aim to combine multiple studies with digital CDT and lipids data for an increased statistical power to detect more significant indirect effects through lipids.

Other limitations included that we did not consider the potential interaction effect between *APOE* and lipids metabolites; future work could explore these interactions to gain deeper insight. Also, because our exposure *APOE* is determined at birth, all confounders in our analysis may potentially act as post-treatment confounders, complicating the causal interpretation. Additionally, all outcomes in this application focus on the CDT time outcomes, which may only reflect certain aspects of cognitive function, for example, processing speed. Our future research also plans to investigate the effects of *APOE* alleles and lipids on different domains of cognitive function using additional measures. Finally, our mediator and outcome models relied on parametric regression approaches. A future direction is to explore the flexible machine learning models to better capture complex relationships in covariates.

## Conclusions

We analyzed data from the LLFS to investigate the relationship between *APOE* variants, lipids, and cognitive function measured by CDT times. The results revealed a direct protective effect of *APOE2* on cognitive and motor function; the results identified indirect effects through several lipid species that mediated the effects of *APOE2* in either protective or deleterious pathways. The identified protective and deleterious lipid pathways present potential opportunities for developing new therapeutics targeting these lipids to modulate the effects of *APOE2* on cognitive function.

## Supplementary Material

Supplementary Material 1

**Supplementary Information** The online version contains supplementary material available at https://doi.org/10.1007/s10654-025-01310-0.

## Figures and Tables

**Table 1:** Characteristics of participants who were included in the analysis

Characteristic	Overall, *N* = 1228	*APOE3*, *N* = 988	*APOE2*, *N* = 240
Age at enrollment	63.0 (57.0, 70.0)	63.0 (57.0, 70.0)	62.0 (56.0, 70.0)
Age at visit two	72.0 (65.0, 79.0)	72.0 (66.0, 79.0)	71.0 (65.0, 80.0)
Sex			
Female	702 (57%)	573 (58%)	129 (54%)
Male	526 (43%)	415 (42%)	111 (46%)
Years of education	14.0 (10.0, 15.0)	14.0 (10.0, 15.0)	14.0 (10.0, 14.0)
BMI	26.8 (24.2, 29.9)	26.8 (24.2, 29.8)	26.9 (24.5, 30.1)
Lipid-lowering medication usage	359 (29%)	317 (32%)	42 (18%)
CDT total-time	32.5 (26.0, 41.9)	33.1 (26.3, 42.3)	30.3 (25.1, 41.0)
Think-time	18.7 (14.0, 25.7)	18.8 (14.1, 25.9)	18.3 (13.9, 24.4)
Ink-time	13.6 (10.8, 16.8)	13.7 (11.0, 16.9)	12.9 (10.0, 16.1)

Continuous variables are summarized with median and interquartile range. Discrete variables are summarized with count and percentage. *APOE3* = e3e3, *APOE2* = e2e2 or e2e3

*BMI* body mass index, *CDT* Clock Drawing Test

**Table 2:** List of 24 lipid species used in the mediation analysis

Standardized name	Super class
CE 18:2	Sterol Lipids
CE 18:3	Sterol Lipids
CE 20:4	Sterol Lipids
CE 22:5	Sterol Lipids
CE 22:6	Sterol Lipids
Cer 33:1	Sphingolipids
DG 38:5	Glycerolipids
dHexCer_NS 34:1	NA
dHexCer_NS 40:1	NA
dHexCer_NS 41:1	NA
dHexCer_NS 42:1	NA
TG 51:0	Glycerolipids
TG 51:3	Glycerolipids
TG 53:1	Glycerolipids
TG 53:2	Glycerolipids
TG 54:1	Glycerolipids
TG 54:2	Glycerolipids
TG 56:1	Glycerolipids
TG 56:2	Glycerolipids
TG 56:3	Glycerolipids
TG 56:4	Glycerolipids
TG 56:5	Glycerolipids
TG 58:3	Glycerolipids
TG 58:6	Glycerolipids

**Table 3:** Primary analysis of estimated indirect effects of *APOE2* on CDT times through each lipid pathway

Indirect effect estimates (95% CI)	Total-time	Think-time	Ink-time
CE 18:2	0.16 (−0.55, 0.96)	0.03 (−0.56, 0.60)	0.14 (−0.11, 0.43)
CE 18:3	−0.23 (−0.56, 0.03)	**−0.20 (−0.46, −0.01)**	−0.02 (−0.13, 0.08)
CE 20:4	−0.47 (−1.20, 0.18)	−0.27 (−0.86, 0.24)	−0.20 (−0.44, 0.01)
CE 22:5	0.22 (−0.21, 0.85)	0.19 (−0.13, 0.67)	0.03 (−0.16, 0.19)
CE 22:6	−0.14 (−0.50, 0.23)	−0.18 (−0.50, 0.11)	0.03 (−0.08, 0.19)
Cer 33:1	−0.16 (−0.60, 0.14)	−0.11 (−0.43, 0.13)	−0.05 (−0.18, 0.06)
DG 38:5	−0.72 (−1.72, 0.12)	−0.47 (−1.14, 0.10)	−0.25 (−0.58, 0.07)
dHexCer_NS 34:1	0.00 (−0.23, 0.16)	0.00 (−0.14, 0.13)	0.00 (−0.08, 0.04)
dHexCer_NS 40:1	−0.06 (−0.86, 0.68)	−0.12 (−0.80, 0.39)	0.05 (−0.17, 0.38)
dHexCer_NS 41:1	0.11 (−0.21, 0.72)	0.10 (−0.14, 0.54)	0.01 (−0.11, 0.18)
dHexCer_NS 42:1	0.09 (−0.81, 1.17)	0.14 (−0.58, 0.95)	−0.04 (−0.41, 0.25)
TG 51:0	−0.18 (−0.88, 0.36)	−0.11 (−0.64, 0.27)	−0.06 (−0.34, 0.13)
TG 51:3	0.40 (−0.21, 1.17)	0.22 (−0.22, 0.76)	0.17 (−0.04, 0.47)
TG 53:1	−0.34 (−1.68, 0.86)	−0.36 (−1.42, 0.52)	0.02 (−0.44, 0.53)
TG 53:2	0.32 (−0.31, 1.20)	0.28 (−0.23, 0.96)	0.04 (−0.19, 0.30)
TG 54:1	0.75 (−0.21, 2.02)	0.57 (−0.11, 1.54)	0.19 (−0.17, 0.67)
TG 54:2	−0.23 (−1.59, 0.97)	−0.22 (−1.27, 0.69)	−0.02 (−0.51, 0.45)
TG 56:1	−1.22 (−3.05, 0.14)	−1.00 (−2.45, 0.06)	−0.23 (−0.79, 0.27)
TG 56:2	1.57 (−0.62, 4.48)	1.58 (−0.12, 4.07)	0.01 (−0.86, 0.85)
TG 56:3	−0.21 (−2.21, 1.46)	−0.09 (−1.44, 1.14)	−0.10 (−0.75, 0.45)
TG 56:4	0.58 (−0.22, 1.57)	0.25 (−0.39, 0.95)	**0.32 (0.03, 0.71)**
TG 56:5	**−1.26 (−3.00, −0.02)**	−0.81 (−2.24, 0.19)	**−0.46 (−1.00, −0.07)**
TG 58:3	−0.21 (−1.40, 1.06)	−0.39 (−1.26, 0.63)	0.16 (−0.22, 0.61)
TG 58:6	0.16 (−0.60, 1.06)	0.08 (−0.49, 0.71)	0.09 (−0.17, 0.35)

95% confidence intervals (CI) are generated using bootstrap percentile method. Protective lipids mediated the effect of *APOE2* to reduce CDT times, while deleterious lipids mediated the effect of *APOE2* to increase CDT times. Bold font indicates that the effect estimate is statistically significant

**Table 4:** Summary of mediation analyses of *APOE2* on the CDT times with 95% CI

	Digital CDT		
	Total-time	Think-time	Ink-time
Primary analysis (95% CI)			
Direct effect	−2.91 (−5.26, −0.59)	−1.90 (−3.60, −0.22)	−0.98 (−1.80, −0.18)
Combined indirect effect	−1.07 (−2.63, 0.58)	−0.89 (−2.28, 0.42)	−0.17 (−0.68, 0.37)
Mediated proportion	27% (−13%, 82%)	32% (−15%, 89%)	15% (−36%, 71%)
Total effect	−3.98 (−6.33, −1.36)	−2.78 (−4.80, −0.69)	−1.15 (−2.02, −0.23)
Secondary analysis (95% CI)			
Direct effect	−3.16 (−5.39, −1.01)	−2.19 (−3.81, −0.64)	−1.06 (−1.88, −0.28)
Combined indirect effect (%)	−0.81 (−1.82, 0.29)	−0.57 (−1.19, 0.06)	−0.09 (−0.31, 0.15)
Mediated proportion	20% (−10%,59%)	21% (−3%, 52%)	8% (−18%, 40%)
Total effect	−3.97 (−6.35, −1.50)	−2.76 (−4.52, −1.02)	−1.15 (−2.01, −0.33)

Negative estimates shows that the *APOE2* carriers has a shorter CDT completion time compared to *APOE3*. Direct effect: the effect of *APOE2* on the CDT time that do not involve lipids. Combined indirect effect: the sum of indirect effects via all lipid pathways. Mediated Proportion: the percentage of the total effect mediated by the combined indirect effects. Total effect: the sum of the direct effect and the combined indirect effect

*CDT* Clock Drawing Test, *CI* confidence interval

**Table 5:** Secondary analysis of indirect effects of *APOE2* on CDT times through each lipid pathway, after variable selection of lipids in the outcome model

Total-time		Think-time		Ink-time	
Lipids	Effect estimate (95% CI)	Lipids	Effect estimate (95% CI)	Lipids	Effect estimate (95% CI)
CE 18:3	−0.19 (−0.48, 0.02)	**CE 18:3**	**−0.17 (−0.38, −0.01)**	**DG 38:5**	**−0.22 (−0.48, 0.00)**
CE 22:6	−0.21 (−0.57, 0.13)	CE 22:6	−0.16 (−0.44, 0.11)	**TG 51:3**	**0.18 (0.02, 0.39)**
Cer 33:1	−0.17 (−0.58, 0.07)	DG 38:5	−0.39 (−0.93, 0.08)	**TG 56:4**	**0.22 (0.04, 0.45)**
DG 38:5	−0.64 (−1.45, 0.11)	**TG 56:1**	**−1.07 (−2.26, −0.18)**	**TG 56:5**	**−0.26 (−0.58, −0.02)**
dHexCer_NS 41:1	0.09 (−0.05, 0.44)	**TG 56:2**	**1.60 (0.68, 3.00)**		
TG 51:3	0.39 (−0.17, 1.02)	TG 56:5	−0.39 (−1.05, 0.05)		
TG 54:1	0.44 (−0.08, 1.12)				
**TG 56:1**	**−1.52 (−3.11, −0.32)**				
**TG 56:2**	**1.41 (0.10, 3.23)**				
TG 56:4	0.48 (−0.10, 1.26)				
**TG 56:5**	**−0.90 (−2.06, −0.14)**				

95% confidence intervals (CI) are generated using bootstrap percentile method. Protective lipids mediated the effect of *APOE2* to reduce CDT times, while deleterious lipids mediated the effect of *APOE2* to increase CDT times. Bold font indicates that the effect estimate is statistically significant
