# Supplementary material for: The role of lipids in the effect of APOE2 on cognitive function: a causal mediation analysis
Source: Eur J Epidemiol. Author manuscript; Available in PMC 2026 Jan 5. (PMC12756338; doi:10.1007/s10654-025-01310-0)
Supplement: Supplementary Material 1 [file NIHMS2128870-supplement-Supplementary_Material_1.docx]

# Supplementary File

### Calculation of direct effects and indirect effects

We provide a detailed description of the computation of direct effects and indirect effects, following the methodology of VanderWeele (2014). We performed a regression approach to estimate the total, direct, and indirect effect of *APOE* on each of the outcomes, total-, ink-, and think-time of the CDT.

Step 1: we fitted the mediator regression models, one for each lipid, denoted as $M^{(i)}$

$$E\left[ log(M^{\left( i \right)}) | a,C \right]=\theta_{0}^{(i)}+\theta_{1}^{(i)}\cdot a_{APOE}+\boldsymbol{\theta}_{\boldsymbol{2}}^{\left( i \right)}\cdot\boldsymbol{C,}$$

where $M^{(i)}$ is the $i$th mediator (lipid), $i=1,2,\ldots,K$*,* $\theta_{0}^{(i)}$ is the intercept, $a_{APOE}$ is the *APOE* genotype group coded as *APOE3*=0, *APOE2*=1 and $\theta_{1}^{(i)}$ is the corresponding coefficient, $\boldsymbol{C}$ is a vector of baseline confounders (sex, education, age at enrollment, and indicator of lipid-lowering medication usage, and indicator of young/old generation) and $\boldsymbol{\theta}_{\boldsymbol{2}}^{\left( i \right)}$ is the vector of their coefficients.

Step 2: we fit the outcome regression model for each outcome, incorporating all lipids simultaneously:

$$E\left[ Y | a,M,C \right]=\beta_{0}+\beta_{1}\cdot a_{APOE}+\beta_{2}^{\left( 1 \right)}\cdot{log(M}^{\left( 1 \right)})+\ldots\beta_{2}^{\left( K \right)}\cdot log(M^{\left( K \right)})+\boldsymbol{\beta}\cdot\boldsymbol{C,}$$

where $Y$ is one of the three CDT times, $\beta_{0}$ is the intercept, $\beta_{1}$, $\beta_{2}^{i}$ and $\boldsymbol{\beta}$ are the corresponding coefficients.

- The direct effect of *APOE2* on the outcome is the estimate of $\beta_{1}$.
- The indirect effect of *APOE2* through the pathway of $i$th lipid metabolite is estimated as the product of the estimates $\hat{\theta}_{1}^{(i)}\cdot\hat{\beta}_{2}^{(i)}.$
- The combined indirect effect of all the lipid metabolites is estimated as $\sum_{i=1}^{K} \hat{\theta}_{1}^{(i)}\cdot\hat{\beta}_{2}^{(i)}$.
- The total effect is the sum of the direct effect and combined indirect effect: $\beta_{1}+\sum_{i=1}^{K} \hat{\theta}_{1}^{(i)}\cdot\hat{\beta}_{2}^{(i)}$.
- The mediated proportion is estimated by the combined indirect effect divided by the total effect: $\sum_{i=1}^{K} \hat{\theta}_{1}^{\left( i \right)}\cdot\hat{\beta}_{2}^{\left( i \right)}/(\beta_{1}+\sum_{i=1}^{K} \hat{\theta}_{1}^{\left( i \right)}\cdot\hat{\beta}_{2}^{\left( i \right)})$

To assess the statistical significance of the estimated direct and indirect effects, we conducted 1500 bootstrap resamples to construct confidence intervals.

### Supplementary Figures


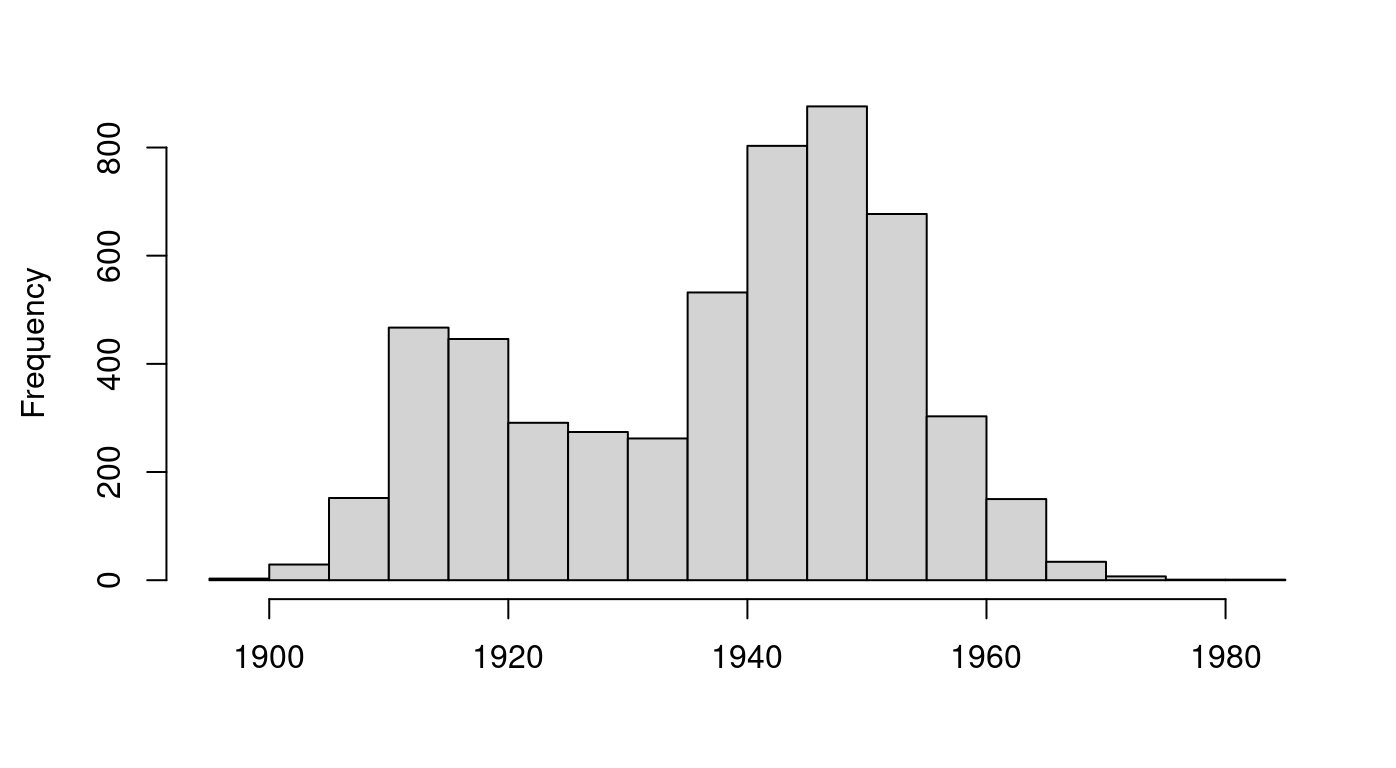


**Supplementary Figure 1**: Date of birth of the LLFS participants. The young/old generation is separated by whether birth year is >= 1935.


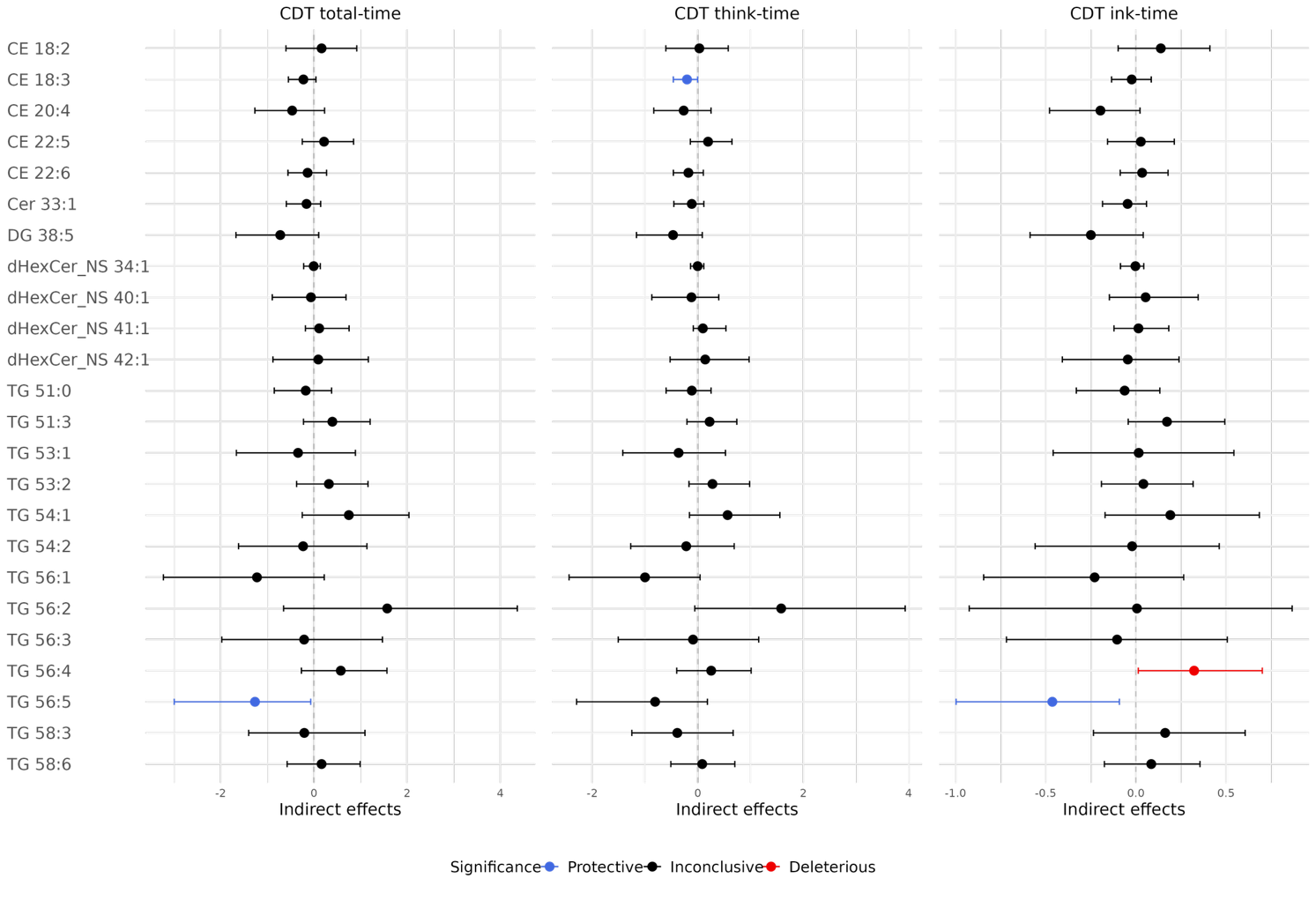
**Supplementary Figure 2**: Sensitivity analysis of indirect effects of *APOE2* on CDT times through each lipid pathway. Stepwise variable selection with AIC criterion was applied in the sensitivity analysis, and hence each CDT time had different lipids remained in the mediation analysis. Blue color indicates statistically significant protective lipids, which mediated the effect of *APOE2* to reduce CDT times. Red color indicates statistically significant deleterious lipids, which mediated the effect of *APOE2* to increase CDT times.

### Supplementary Tables: primary analysis results of mediator regression and outcome regression

| Standardized Name | Super Class | Adjusted p-value | Estimated effect |
| --- | --- | --- | --- |
| CE 18:2 | Sterol Lipids | 7.34E-06 | -0.027 |
| CE 18:3 | Sterol Lipids | 3.52E-02 | -0.062 |
| CE 20:4 | Sterol Lipids | 1.15E-05 | -0.08 |
| CE 22:5 | Sterol Lipids | 2.38E-02 | -0.077 |
| CE 22:6 | Sterol Lipids | 4.87E-05 | -0.159 |
| Cer 33:1 | Sphingolipids | 6.93E-03 | -0.066 |
| DG 38:5 | Glycerolipids | 1.36E-04 | 0.155 |
| dHexCer_NS 34:1 | NA | 2.38E-02 | -0.057 |
| dHexCer_NS 40:1 | NA | 4.16E-02 | -0.063 |
| dHexCer_NS 41:1 | NA | 1.76E-02 | -0.07 |
| dHexCer_NS 42:1 | NA | 4.37E-03 | -0.081 |
| TG 51:0 | Glycerolipids | 4.46E-02 | 0.159 |
| TG 51:3 | Glycerolipids | 4.08E-02 | -0.048 |
| TG 53:1 | Glycerolipids | 4.38E-05 | 0.231 |
| TG 53:2 | Glycerolipids | 3.55E-02 | 0.092 |
| TG 54:1 | Glycerolipids | 1.35E-03 | 0.158 |
| TG 54:2 | Glycerolipids | 2.17E-03 | 0.096 |
| TG 56:1 | Glycerolipids | 2.77E-08 | 0.331 |
| TG 56:2 | Glycerolipids | 9.47E-07 | 0.24 |
| TG 56:3 | Glycerolipids | 1.19E-05 | 0.144 |
| TG 56:4 | Glycerolipids | 6.11E-03 | 0.09 |
| TG 56:5 | Glycerolipids | 8.13E-03 | 0.066 |
| TG 58:3 | Glycerolipids | 6.37E-10 | 0.272 |
| TG 58:6 | Glycerolipids | 4.47E-04 | 0.103 |

**Supplementary Table 1**: Total 24 lipids included in this analysis. The association between APOE2 and each lipid was estimated using regression analysis with p-value adjusted 5% False Discovery Rate (FDR). This table is adapted from the Supplementary Figure 2 in Sebastiani et al. (2024).

| Lipid (log scale) | $\boldsymbol{\beta}$ of *APOE3 vs APOE2* (SD) | P-value |
| --- | --- | --- |
| CE 18:2 | -0.35 (0.07) | <0.01* |
| CE 18:3 | -0.19 (0.06) | <0.01* |
| CE 20:4 | -0.28 (0.07) | <0.01* |
| CE 22:5 | -0.23 (0.06) | <0.01* |
| CE 22:6 | -0.19 (0.07) | <0.01* |
| Cer 33:1 | -0.19 (0.07) | <0.01* |
| DG 38:5 | 0.35 (0.06) | <0.01* |
| dHexCer_NS 34:1 | -0.02 (0.06) | 0.74 |
| dHexCer_NS 40:1 | -0.13 (0.07) | 0.06 |
| dHexCer_NS 41:1 | -0.08 (0.06) | 0.21 |
| dHexCer_NS 42:1 | -0.16 (0.06) | <0.01* |
| TG 51:0 | 0.14 (0.07) | <0.01* |
| TG 51:3 | -0.30 (0.07) | 0.03* |
| TG 53:1 | 0.27 (0.07) | <0.01* |
| TG 53:2 | 0.17 (0.07) | <0.01* |
| TG 54:1 | 0.23 (0.06) | <0.01* |
| TG 54:2 | 0.25 (0.07) | <0.01* |
| TG 56:1 | 0.34 (0.07) | <0.01* |
| TG 56:2 | 0.36 (0.07) | <0.01* |
| TG 56:3 | 0.38 (0.08) | <0.01* |
| TG 56:4 | 0.30 (0.07) | <0.01* |
| TG 56:5 | 0.29 (0.07) | <0.01* |
| TG 58:3 | 0.41 (0.07) | <0.01* |

**Supplementary Table 2**: Results of the mediator regression in the primary analysis. Dependent variable: lipids (log scale and standardized). Independent variables: *APOE3* (reference group) vs *APOE2*, age at enrollment, sex, education, BMI, lipid-lowering medication usage, and indicator for young/old generation. The column names correspond to lipids, the dependent variable. $\beta$: estimated coefficients of *APOE3* (reference) versus *APOE2* on each lipid. SD: standard deviation. *: p-value that reaches the significance level of 0.05.

|  | Total-time | | Think-time | | Ink-time | |
| --- | --- | --- | --- | --- | --- | --- |
|  | $\beta$ (SD) | p-value | $\beta$ (SD) | p-value | $\beta$ (SD) | p-value |
| *APOE2* | **-2.91 (1.03)** | **<0.01** | **-1.90 (0.75)** | **<0.01** | **-0.98 (0.41)** | **<0.01** |
| CE 18:2 | -0.46 (0.9) | 0.61 | -0.08 (0.68) | 0.90 | -0.39 (0.33) | 0.24 |
| CE 18:3 | 1.18 (0.67) | 0.08 | **1.07 (0.49)** | **0.03** | 0.12 (0.25) | 0.63 |
| CE 20:4 | 1.64 (1.13) | 0.15 | 0.94 (0.87) | 0.28 | 0.69 (0.38) | 0.07 |
| CE 22:5 | -0.95 (0.97) | 0.33 | -0.85 (0.73) | 0.24 | -0.12 (0.36) | 0.73 |
| CE 22:6 | 0.72 (0.96) | 0.45 | 0.94 (0.74) | 0.20 | -0.18 (0.32) | 0.56 |
| Cer 33:1 | 0.86 (0.70) | 0.22 | 0.61 (0.55) | 0.27 | 0.25 (0.23) | 0.27 |
| DG 38:5 | -2.07 (1.18) | 0.08 | -1.35 (0.84) | 0.11 | -0.72 (0.41) | 0.08 |
| dHexCer_NS 34:1 | 0.24 (0.85) | 0.78 | 0.12 (0.65) | 0.85 | 0.11 (0.29) | 0.70 |
| dHexCer_NS 40:1 | 0.51 (2.33) | 0.83 | 0.95 (1.74) | 0.58 | -0.43 (0.75) | 0.57 |
| dHexCer_NS 41:1 | -1.46 (1.40) | 0.30 | -1.25 (1.05) | 0.23 | -0.19 (0.47) | 0.69 |
| dHexCer_NS 42:1 | -0.58 (2.58) | 0.82 | -0.88 (1.88) | 0.64 | 0.28 (0.83) | 0.74 |
| TG 51:0 | -1.22 (1.60) | 0.45 | -0.78 (1.18) | 0.51 | -0.43 (0.59) | 0.47 |
| TG 51:3 | -1.34 (0.96) | 0.16 | -0.76 (0.69) | 0.27 | -0.58 (0.35) | 0.10 |
| TG 53:1 | -1.28 (2.16) | 0.55 | -1.36 (1.6) | 0.40 | 0.06 (0.77) | 0.94 |
| TG 53:2 | 1.94 (1.84) | 0.29 | 1.69 (1.37) | 0.22 | 0.25 (0.63) | 0.69 |
| TG 54:1 | 3.28 (2.06) | 0.11 | 2.47 (1.49) | 0.10 | 0.84 (0.79) | 0.29 |
| TG 54:2 | -0.92 (2.24) | 0.68 | -0.87 (1.63) | 0.60 | -0.08 (0.83) | 0.92 |
| TG 56:1 | -3.6 (2.12) | 0.09 | -2.94 (1.55) | 0.06 | -0.67 (0.75) | 0.37 |
| TG 56:2 | 4.33 (3.00) | 0.15 | 4.35 (2.31) | 0.06 | 0.02 (1.05) | 0.99 |
| TG 56:3 | -0.56 (2.17) | 0.80 | -0.24 (1.59) | 0.88 | -0.28 (0.78) | 0.72 |
| TG 56:4 | 1.89 (1.29) | 0.14 | 0.84 (0.96) | 0.38 | **1.06 (0.49)** | **0.03** |
| TG 56:5 | **-4.37 (2.27)** | **0.05** | -2.79 (1.83) | 0.13 | **-1.60 (0.60)** | **0.01** |
| TG 58:3 | -0.5 (1.31) | 0.70 | -0.94 (0.99) | 0.34 | 0.39 (0.43) | 0.36 |
| TG 58:6 | 0.45 (0.97) | 0.64 | 0.23 (0.76) | 0.76 | 0.24 (0.33) | 0.47 |

**Supplementary Table 3**: Results of the outcome regression in the primary analysis of digital CDT times. Dependent variable: Digital CDT total-time, ink-time, and think-time. Independent variables: *APOE3* (reference group) vs *APOE2*, lipids (log scale and standardized), age at enrollment, sex, education, BMI, lipid-lowering medication usage, and indicator of young/old generation. The column names correspond to *APOE2* and lipids, the independent variable. $\beta$: estimated coefficients for the independent variable. SD: standard deviation. Bold font indicates the estimated coefficients reaching the significance level of 0.05.

### Supplementary Tables: secondary analysis after variable selection of lipids

| Total-time | | | Think-time | | | Ink-time | | |
| --- | --- | --- | --- | --- | --- | --- | --- | --- |
| Lipids | $\beta$ of *APOE3 vs APOE2* (SD) | P-value | Lipids | $\beta$ of *APOE3 vs APOE2* (SD) | P-value | Lipids | $\beta$ of *APOE3 vs APOE2* (SD) | P-value |
| CE 18:3 | -0.2 (0.06) | <0.01* | CE 18:3 | -0.20 (0.06) | <0.01* | DG 38:5 | 0.37 (0.06) | <0.01* |
| CE 22:6 | -0.22 (0.07) | <0.01* | CE 22:6 | -0.22 (0.07) | <0.01* | TG 51:3 | -0.29 (0.07) | <0.01* |
| Cer 33:1 | -0.16 (0.07) | <0.01* | DG 38:5 | 0.37 (0.06) | <0.01* | TG 56:4 | 0.29 (0.07) | <0.01* |
| DG 38:5 | 0.37 (0.06) | <0.01* | TG 56:1 | 0.38 (0.08) | <0.01* | TG 56:5 | 0.29 (0.07) | <0.01* |
| dHexCer_NS 41:1 | -0.09 (0.06) | 0.13 | TG 56:2 | 0.38 (0.07) | <0.01* |  |  |  |
| TG 51:3 | -0.29 (0.07) | <0.01* | TG 56:5 | 0.29 (0.07) | <0.01* |  |  |  |
| TG 54:1 | 0.26 (0.07) | <0.01* |  |  |  |  |  |  |
| TG 56:1 | 0.38 (0.08) | <0.01* |  |  |  |  |  |  |
| TG 56:2 | 0.38 (0.07) | <0.01* |  |  |  |  |  |  |
| TG 56:4 | 0.29 (0.07) | <0.01* |  |  |  |  |  |  |
| TG 56:5 | 0.29 (0.07) | <0.01* |  |  |  |  |  |  |

**Supplementary Table 4**: Results of the mediator regression in the secondary analysis on CDT times. After variable selection in the secondary analysis, less lipids remained in the model. $\beta$: estimated coefficients of E2 versus E3 (reference) on each lipid outcome. SD: standard deviation. *: p-value that reaches the significance level of 0.05.

| Total-time | | | Think-time | | | Ink-time | | |
| --- | --- | --- | --- | --- | --- | --- | --- | --- |
| Variables | $\beta$ (SD) | P-value | Variables | $\beta$ (SD) | P-value | Variables | $\beta$ (SD) | P-value |
| ***APOE2*** | **-3.13 (0.96)** | **<0.01*** | ***APOE2*** | **-2.10 (0.72)** | **<0.01** | ***APOE2*** | **-1.16 (0.38)** | **<0.01*** |
| CE 18:3 | 1.09 (0.57) | 0.06 | CE 18:3 | **0.90 (0.37)** | **0.02** | DG 38:5 | -0.49 (0.28) | 0.09 |
| CE 22:6 | 1.01 (0.8) | 0.20 | CE 22:6 | 0.74 (0.58) | 0.20 | **TG 51:3** | **-0.60 (0.24)** | **0.01** |
| Cer 33:1 | 0.95 (0.55) | 0.09 | DG 38:5 | -1.08 (0.64) | 0.09 | **TG 56:4** | **0.77 (0.29)** | **<0.01*** |
| DG 38:5 | -1.71 (0.99) | 0.08 | TG 56:1 | **-3.20 (1.21)** | **<0.01*** | **TG 56:5** | **-1.01 (0.4)** | **0.01** |
| dHexCer_NS 41:1 | -1.01 (0.64) | 0.11 | TG 56:2 | **4.59 (1.21)** | **<0.01*** |  |  |  |
| TG 51:3 | -1.37 (0.82) | 0.10 | TG 56:5 | **-1.37 (0.86)** | **0.11** |  |  |  |
| TG 54:1 | 1.88 (1.01) | 0.06 |  |  |  |  |  |  |
| **TG 56:1** | **-4.60 (1.66)** | **<0.01*** |  |  |  |  |  |  |
| **TG 56:2** | **4.15 (1.7)** | **0.01*** |  |  |  |  |  |  |
| TG 56:4 | 1.71 (0.92) | 0.06 |  |  |  |  |  |  |
| **TG 56:5** | **-3.30 (1.32)** | **0.01*** |  |  |  |  |  |  |

**Supplementary Table 5**: Results of the outcome regression in the secondary analysis on CDT times. After variable selection in the secondary analysis, less lipids remained in the model. $\beta$: estimated coefficients. SD: standard deviation. Bold font indicates the estimated coefficient reaching the significance level of 0.05.

### Supplementary Tables: sensitivity analysis of digital CDT after excluding the variable of lipid-lowering medication usage

| Indirect effect estimates (95% CI) | Total-time | Think-time | Ink-time |
| --- | --- | --- | --- |
| CE 18:2 | 0.16 (-0.60, 0.92) | 0.03 (-0.60, 0.58) | 0.14 (-0.10, 0.41) |
| CE 18:3 | -0.23 (-0.55, 0.04) | **-0.20 (-0.46, 0.01)** | -0.02 (-0.13, 0.09) |
| CE 20:4 | -0.47 (-1.27, 0.23) | -0.27 (-0.83, 0.25) | -0.20 (-0.48, 0.02) |
| CE 22:5 | 0.22 (-0.25, 0.85) | 0.19 (-0.14, 0.65) | 0.03 (-0.16, 0.21) |
| CE 22:6 | -0.14 (-0.56, 0.27) | -0.18 (-0.46, 0.11) | 0.03 (-0.09, 0.18) |
| Cer 33:1 | -0.16 (-0.59, 0.14) | -0.11 (-0.45, 0.11) | -0.05 (-0.18, 0.06) |
| DG 38:5 | -0.72 (-1.67, 0.10) | -0.47 (-1.16, 0.09) | -0.25 (-0.59, 0.04) |
| dHexCer_NS 34:1 | 0.00 (-0.22, 0.14) | 0.00 (-0.14, 0.11) | 0.00 (-0.09, 0.04) |
| dHexCer_NS 40:1 | -0.06 (-0.89, 0.69) | -0.12 (-0.87, 0.40) | 0.05 (-0.15, 0.35) |
| dHexCer_NS 41:1 | 0.11 (-0.18, 0.75) | 0.10 (-0.08, 0.53) | 0.01 (-0.12, 0.18) |
| dHexCer_NS 42:1 | 0.09 (-0.88, 1.17) | 0.14 (-0.52, 0.97) | -0.04 (-0.41, 0.24) |
| TG 51:0 | -0.18 (-0.85, 0.38) | -0.11 (-0.60, 0.25) | -0.06 (-0.33, 0.13) |
| TG 51:3 | 0.40 (-0.22, 1.21) | 0.22 (-0.20, 0.74) | 0.17 (-0.04, 0.49) |
| TG 53:1 | -0.34 (-1.66, 0.89) | -0.36 (-1.42, 0.53) | 0.02 (-0.46, 0.54) |
| TG 53:2 | 0.32 (-0.37, 1.16) | 0.28 (-0.17, 0.98) | 0.04 (-0.19, 0.32) |
| TG 54:1 | 0.75 (-0.25, 2.04) | 0.57 (-0.16, 1.56) | 0.19 (-0.17, 0.69) |
| TG 54:2 | -0.23 (-1.62, 1.14) | -0.22 (-1.27, 0.69) | -0.02 (-0.56, 0.46) |
| TG 56:1 | -1.22 (-3.23, 0.22) | -1.00 (-2.44, 0.04) | -0.23 (-0.84, 0.27) |
| TG 56:2 | 1.57 (-0.65, 4.36) | 1.58 (-0.06, 3.93) | 0.01 (-0.92, 0.87) |
| TG 56:3 | -0.21 (-1.98, 1.47) | -0.09 (-1.51, 1.16) | -0.10 (-0.72, 0.51) |
| TG 56:4 | 0.58 (-0.27, 1.57) | 0.25 (-0.40, 1.01) | **0.32 (0.01, 0.70)** |
| TG 56:5 | **-1.26 (-3.00, -0.07)** | -0.81 (-2.30, 0.18) | **-0.46 (-1.00, -0.09)** |
| TG 58:3 | -0.21 (-1.40, 1.10) | -0.39 (-1.25, 0.67) | 0.16 (-0.24, 0.61) |
| TG 58:6 | 0.16 (-0.58, 0.99) | 0.08 (-0.51, 0.70) | 0.09 (-0.17, 0.36) |

**Supplementary Table 6**: Results of estimated indirect effects of *APOE2* on CDT times through each lipid pathway after excluding the lipid-lowering medication use variable. 95% confidence intervals (CI) are generated using bootstrap percentile method. Protective lipids mediated the effect of *APOE2* to reduce CDT times, while deleterious lipids mediated the effect of *APOE2* to increase CDT times. Bold font indicates that the effect estimate is statistically significant.
